# Acquired Hemophilia A With Post‐Phlebotomy Compartment Syndrome

**DOI:** 10.1002/ccr3.71360

**Published:** 2025-10-27

**Authors:** Wing Kit Lam, Yuen Ting Sin, Keith Ka Wai Wong

**Affiliations:** ^1^ Department of Clinical Pathology Tuen Mun Hospital New Territories Hong Kong; ^2^ Department of Medicine and Geriatrics Tuen Mun Hospital New Territories Hong Kong

**Keywords:** bleeding, coagulation factor, compartment syndromes, hemophilia

## Abstract

Acquired hemophilia A should be considered in unexplained bleeding with isolated activated partial thromboplastin time prolongation, even if mild. This case demonstrates an atypical presentation—compartment syndrome post‐phlebotomy—with refractory bleeding due to factor VIII inhibitors. High clinical suspicion is essential for early diagnosis and management.

A 63‐year‐old man presented to the emergency department with progressive left upper limb swelling and pain 4 days after phlebotomy at the left cubital fossa for investigation of intermittent bruising over the jaw, right wrist, and left groin. On physical examination, his forearm was tender with tense skin, bruises, and multiple blisters over the volar side of the left forearm (Figure 1A). The left radial pulse is still present with distal sensation intact. Blood tests showed a hemoglobin level of 8.8 g/dL, a normal platelet count, and an isolated prolongation of activated partial thromboplastin time (APTT) at 51.5 s (reference interval: 23.0–34.7 s).

**FIGURE 1:**
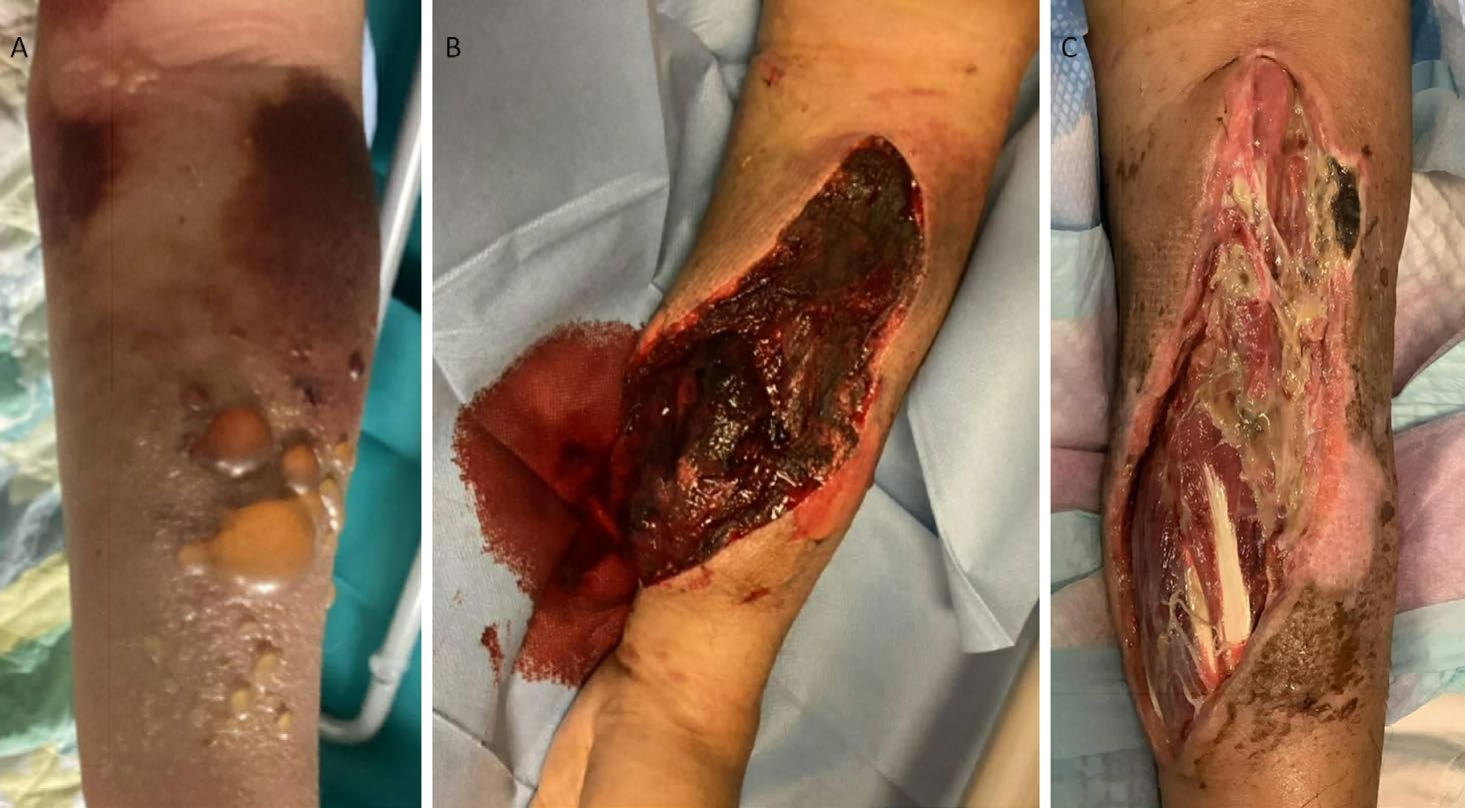
Images of the left forearm in this patient with acquired hemophilia A. (A) Initial presentation with compartment syndrome showing tense skin, bruises, and blisters. (B) Diffuse wound bleeding after fasciotomy. (C) Resolution of bleeding after immunosuppressive therapy.

Given the clinical suspicion of compartment syndrome, the patient was admitted to the orthopedics ward and underwent emergency fasciotomy. Intraoperatively, diffuse bleeding was observed from the subcutaneous tissues, dermis, and muscles. Although initial hemostasis was achieved after platelet and plasma transfusions, diffuse wound bleeding recurred on postoperative day 3 (Figure 1B). The APTT remained prolonged despite repeated plasma transfusions.

This persistent, unexplained bleeding prompted an urgent hematology consultation. Empirical factor eight inhibitor bypassing agent (FEIBA) was initiated for bleeding control while a thorough investigation for an underlying bleeding disorder was undertaken. Factor inhibitor screening showed a time‐ and temperature‐dependent inhibition of the APTT. Factor VIII activity was markedly reduced at 1.3%, and a Bethesda assay confirmed the presence of a factor VIII inhibitor at a level of 14.8 Bethesda units per milliliter, establishing the diagnosis of acquired Hemophilia A (AHA). The patient was subsequently started on immunosuppressive therapy, resulting in resolution of the bleeding (Figure 1C) and normalization of APTT within 10 days.

AHA is a rare bleeding disorder caused by autoantibodies against Factor VIII, most commonly presenting with mucocutaneous bleeding [1]. Diagnosing AHA can be challenging and is often delayed due to a lack of awareness [2]. This case showed an atypical presentation with compartment syndrome following phlebotomy, a seemingly innocuous procedure. The only initial laboratory abnormality was a modestly prolonged APTT, which could easily be overlooked or attributed to other causes. The persistent, refractory bleeding ultimately prompted the hematology consultation that led to the targeted investigation and diagnosis. This case highlights the importance of considering AHA in the differential diagnosis of patients with unexplained or excessive bleeding even when the APTT prolongation is only modest. A high index of suspicion is important for prompt diagnosis and appropriate management.

## Author Contributions

**Wing Kit Lam:** conceptualization, formal analysis, investigation, methodology, visualization, writing – original draft, writing – review and editing. **Yuen Ting Sin:** investigation, writing – review and editing. **Keith Ka Wai Wong:** investigation, writing – review and editing.

## Ethics Statement

The authors have nothing to report.

## Consent

Written informed consent has been obtained from the patient for this publication.

## Conflicts of Interest

The authors declare no conflicts of interest.

## Data Availability

Data sharing is not applicable to this article as no new data were created.
